# Copper surfaces in the ICU reduced the relative risk of acquiring an infection while hospitalized

**DOI:** 10.1186/1753-6561-5-S6-O53

**Published:** 2011-06-29

**Authors:** MG Schmidt, Copper Touch Surface Initiative

**Affiliations:** 1 Microbiology and Immunology, Medical University of South Carolina, Charleston, USA

## Introduction / objectives

The acquisition of microbes with the subsequent development of an infection while hospitalized continues to challenge healthcare worldwide. The CDC estimates the overall risk, mortality and cost to the USA to be ~5%, 100,000 deaths and ~45 billion additional dollars; rates for Medical Intensive Care Units (MICU) are higher where the risk often exceeds 25%. At issue is whether reducing the microbial burden of the environment can lead to an effective method to limit the risk of acquiring an infection while hospitalized.

## Methods

A multi-site clinical trial was conducted within the MICU of 3 US hospitals. The study addressed two issues. First, the effectiveness with which antimicrobial copper touch surfaces would lower the microbial burden found on commonly touched objects and secondly whether a reduction to burden would mitigate the acquisition of an infection while being treated in rooms with copper objects. Microbial burden was assessed in experimental and control rooms once each week. 650 patients were evaluated in order to address whether the presence of copper objects in the room had an impact on the rate of MRSA and/or VRE colonization/infection and/or HAI acquisitions according to the surveillance definition for acute care settings of the CDC/NHSN.

## Results

The median burden observed on copper surfaces was 97% less than the control surfaces which concomitantly resulted in a significant reduction to the number of infections seen in patients treated in copper rooms.

## Conclusion

Use of antimicrobial copper surfaces facilitated a reduction in burden to levels below those suggested for the terminal-cleaning standard of 5 cfu/cm^2^. Risk mitigation of the environmental burden resulted in a concomitant mitigation of the HAI rates for patients treated in rooms with selected high impact touch surfaces fabricated from antimicrobial copper.

## Disclosure of interest

M. Schmidt Grant/Research support from US Army Medical Research & Materiel Command under Contract W81XWH-07-C-0053.

